# Cross-Wavelet Time-Frequency Analysis Reveals Sympathetic Contribution to Baroreflex Sensitivity as Cause of Variable Phase Delay Between Blood Pressure and Heart Rate

**DOI:** 10.3389/fnins.2019.00694

**Published:** 2019-07-09

**Authors:** Roel W. de Boer, John M. Karemaker

**Affiliations:** Department of Medical Biology, Section Systems Physiology, Amsterdam University Medical Centers, Location AMC, Amsterdam, Netherlands

**Keywords:** baroreflex sensitivity, cross-wavelet analysis, xBRS, phase delay, cardiovascular variability, finger blood pressure, heart rate, blood pressure

## Abstract

**Introduction:**

Baroreflex sensitivity (BRS) is often presented as a single number, but it is actually a frequency-dependent phenomenon whose value changes constantly due to internal and external stimuli. The standing posture, for instance, necessitates a changeover from vagal to sympathetic predominance for cardiovascular control. We present a wavelet cross-spectral analysis of blood pressure (BP) and interbeat interval (IBI) recordings in the search for variations in gain and phase between these signals. Additionally, we show how the lag in sympathetic response dictates BP-to-IBI phase relations.

**Methods:**

Recordings in supine and head-up tilted (HUT) position, obtained earlier in 10 healthy subjects (4f/6m, aged 27–47 years) were used. BP and IBI were measured from the continuous finger pressure (by Finometer). The cross-wavelet analysis produced time- and frequency dependent gain (wBRS, wavelet derived BRS) and phase, using the MATLAB^®^ wavelet toolbox. We also applied the wBRS method to model-generated BP- and IBI-data with known interrelations to test the results of this analysis technique. Finally, wBRS values were compared with the xBRS-approach, which is a time domain method for continuous BRS estimation in a sliding 10-s window.

**Results:**

In resting supine conditions, wBRS fluctuates; more at respiratory frequencies than in the 0.1 Hz band. After HUT, wBRS at the respiratory frequency decreases from average 22.7 to 8.5 ms/mmHg, phase between BP and IBI increases from −30° to −54°; in the sympathetic 0.1 Hz range these numbers are 13.3→6.3 ms/mmHg and −54°→−59°. The values found by xBRS are intermediate between wBRS-resp and wBRS-0.1 Hz. The Appendix shows that for the simulated data the BRS and phase values as found by the wavelet technique can be explained from vector additions of vagal and sympathetic BRS contributions.

**Discussion:**

During supine rest parasympathetic control of heart rate dominates BRS; after HUT this is diminished and less effective. Due to the reaction times of the autonomic effectors, the phase relations between the signals depend on the relative contribution of the sympathetics, which explains the larger phase shift.

**Conclusion:**

Cross wavelet analysis allows to follow fast BRS changes in time and frequency, while the computed phase relations help understand sympathetic participation.

## Introduction

Baroreflex sensitivity (BRS) is commonly defined as the ratio of a change in inter beat-interval (IBI) and the change in systolic arterial blood pressure (SAP) that caused it: BRS = ΔIBI/ΔSAP. Several techniques have been proposed for the determination of the BRS, e.g., by studying the resulting IBI-increase after an angiotensin-provoked rise of blood pressure (Smyth et al., 1969), or following neck suction or a Valsalva maneuver (Goldstein et al., 1982). Later, investigators derived BRS estimators from spontaneous fluctuations in pressure and heart-rate for subjects in resting conditions, either using time-domain techniques such as sequential methods (Bertinieri et al., 1985; Parati et al., 1988), or applying spectral approaches (De Boer et al., 1986; Robbe et al., 1987; Parati et al., 1995).

Most of these techniques return a single BRS-value over a period of time, although time-varying BRS-values have been considered, both for time-domain (Westerhof et al., 2004; Eckberg and Kuusela, 2005; Wesseling et al., 2017) and frequency domain (e.g., Li et al., 2018) approaches. Several authors used transfer function analysis to obtain frequency-dependent BRS values, mainly in animal models; a review is given by Kawada and Sugimachi (2016).

In real life, the value of the BRS will change continuously, due to internal and external stimuli, of which a change of posture is a very strong one, as it necessitates activation of the sympathetic nervous outflow to the vasculature and concomitantly to the heart, thereby increasing heart rate and contractility, although that effect is not strictly necessary as is proven in patients who have a cardiac transplant (Rudas et al., 1993).

**FIGURE 1:**
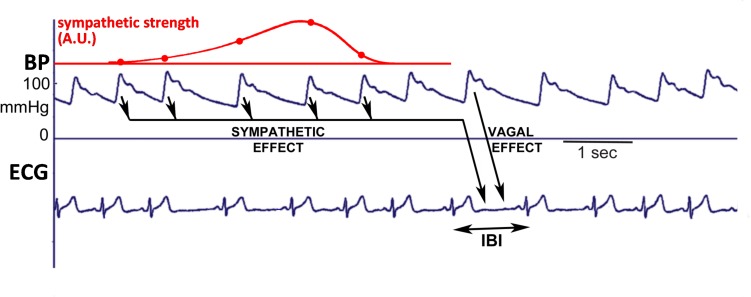
Schematic diagram to explain how the simulated IBI-data were generated. The systolic pressure is considered to rapidly affect the length of the ongoing IBI, due to the vagal effect. In addition, the values of diastoles/systoles activate the sympathetics: lower pressure values cause more activity, inducing a shorter IBI. After a pressure change, the effect on IBI starts slowly, but lasts for some seconds, and so the considered IBI is affected by several past pressure-values, as depicted by the arrows. The dots on the red line indicate the relative contributions of each of these previous pressure values to the ongoing beat duration, in addition to the vagal effect.

Figure 1 presents a schematic diagram of presumed baroreflex-mediated delays between blood pressure (BP) variations and IBI variations. A change in BP affects the heart rate first of all by fast vagal influence, which may affect the very interval during which the systolic pressure happens, or the next one (Pickering and Davies, 1973). In addition, the slower sympathetic effect of BP variations on IBI is observed only after a delay of some 2–3 s (Borst and Karemaker, 1983) and so the length of the present IBI is affected both by the value of the present BP and by the combined effect of a number of previous BP values. Hence, for a slowly varying pressure this delay in the sympathetic contribution to the BRS may counteract the expected effect. A simple example, considering an open-loop situation: if the sympathetic influence would have a fixed time to peak effect of 4 s, then its action on 8-s variations in blood-pressure (0.125 Hz) would be counter-intuitive: during an increase in BP the sympathetic effect would tend to increase the heart rate. The vagal action would still work to lower the heart rate under these conditions and so an apparent *negative* sympathetic contribution to the total BRS-value appears. It follows that the observed BRS is a combination of vagal and sympathetic effects, which leads to a frequency-dependent phase-difference between pressure- and interval variations, as shown previously (De Boer et al., 1987). In the present study we will stress that BRS should not be considered as one number, but as a variable, frequency-dependent phenomenon.

To study simultaneously the variation in time and the spectral properties of the BRS, we applied a cross wavelet analysis technique. We tested its use on a set of experimental data which were available from earlier experiments in our laboratory (JMK). Data from 10 healthy subjects were analyzed; they were relaxed, supine (supposedly in a vagus-dominated state), then had three periods of paced breathing, followed by a head-up tilt, which would lead to a sympathetically dominated state. We applied wavelet cross-spectral analysis, which revealed variable gain and phase in the computed BRS during the experimental protocol. Similar techniques have been used by Kashihara et al. (2009) for data from anesthetized rabbits and by Keissar et al. (2010), both for normal subjects and for patients with cardiovascular issues. For comparison, we also show an analysis of the variability of the BRS as found by the xBRS technique for the considered data (Westerhof et al., 2004; Wesseling et al., 2017).

In addition, we performed the same spectral analysis for a set of simulated BP and IBI data. The simulated BP values are varying with both 0.1 Hz and a respiratory frequency (0.25 Hz). The BP data generate IBI values, using a simple model for the vagal and sympathetic influence on heart rate. The analysis of the simulated data both corroborates the validity of the applied analysis technique, and clarifies the differences in computed wavelet-derived BRS values (wBRS) under different circumstances.

## Materials and Methods

### Subjects, Experimental Conditions

We used a subset of 25-min recordings from an experimental study into orthostatic tolerance performed in our Institute in the period 2001–2002 under auspices of the European Space Agency (ESA). The study had been authorized by the appropriate Ethical Boards and subjects had given written informed consent in accordance with the Declaration of Helsinki. The anonymized data from 10 subjects (4f/6m) were used, average age 35 years (range 27–47), BMI 21.9 kg/m^2^ (19.3–26.0). The subjects were resting supine on a tilt table for 10 min, followed by three frequencies of paced breathing (audio cue, 10, 6, 15/min, respectively, each for 1.2 min, 1 min recovery); rate and hyper- or hypoventilation were checked by continuous measurement of expired CO_2_ level. Then subjects were head-up tilted (HUT) in 1 s to a 70°, relaxed standing position, remained leaning against the table for 5 min and finally they were tilted back and 2 min of relaxed supine recording followed (Gisolf et al., 2004). In this study we used the IBI data and the derived systolic pressures from the continuous finger blood pressure data (Finometer, BMI-TNO, Netherlands; sample frequency 200 Hz, start of an IBI is set by the firmware at a point that corresponds to the very start of the systolic upslope as determined by a proprietary algorithm).

The data were analyzed using the beat-to-beat formalism, in which the *n*th systolic pressure (SAP*_*n*_*) gets the same index as the IBI in which it occurs (IBI*_*n*_*), and for spectral analysis purposes the time between items of the series (i.e., the sample frequency) is set to equal the mean IBI (De Boer et al., 1984, 1985). This is the most unambiguous way to study time- and phase-relationships between blood-pressure and IBI data (Karemaker and De Boer, 2017).

### Simulated Data

Simulated data were constructed that are similar to observed data from our experimental protocol. A series of 2000 SAP-IBI pairs (“heart beats”) was prepared. The pressure values consisted of a mean value of 120 mmHg plus the sum of two time-dependent sinusoidal contributions with frequencies of 0.1 and 0.25 Hz and amplitude 5 mmHg each. In addition, Gaussian noise (sigma = 2 mmHg) was added. The baroreflex control of IBI by the systolic pressures is modeled by vagal and sympathetic contributions (Figure 1). To relate the simulated data to the experimental protocol, the mean IBI and the vagal strength were set at different values during the simulated “supine” period (the first 1500 and final 200 beats) than during the “head up tilt” period (beats 1500–1800). Mean IBI was set at 1000 ms (supine) and 700 ms (HUT). Fast (“vagal”) and slower (“sympathetic”) baroreflex contributions transformed the fluctuations in pressure values into IBI-variations. The vagal BRS, affecting the length of the very interval in which the systolic pressure occurred, was set at 9 ms/mmHg in the supine period and at 3 ms/mmHg during HUT. The sympathetic contribution consisted of a time-varying contribution of previous pressures, linearly increasing from zero to 3 ms/mmHg between 5.6 and 3.2 s before the considered IBI, and next decreasing to zero again at 0.8 s. Gaussian noise (sigma = 5 ms) was added. The parameters we used are taken from our 1987 paper (De Boer et al., 1987), where a justification for their values is given.

### Analysis Technique

Wavelets are short oscillatory signals with an amplitude that goes from zero to a maximum and back to zero; wavelets are characterized by their shape, frequency and duration (Torrence and Compo, 1998). To apply wavelet analysis to a signal, e.g., a cardiovascular signal, the wavelet is convolved with the signal. A high correlation value at a certain point in time implies that the signal at that moment contains information at the frequency of the wavelet. By applying a series of wavelets with different frequencies to the signal, its frequency content at each moment in time can be determined. This is in contrast to standard Fourier-analysis techniques, which compute the frequency content of the signal over a period in time.

Cross wavelet analysis is a technique that was developed in the 1980s for the simultaneous analysis of two signals in the frequency domain and in the time domain. It is mainly used in fields such as oceanography (Jevrejeva et al., 2003), meteorology (Torrence and Compo, 1998), and econometrics (Rua and Nunes, 2009). The technique has also been applied for studies in circulation physiology (Kashihara et al., 2009; Keissar et al., 2010). The great strength of cross analysis is that it enables one to study how spectral features evolve over time. Hence values for magnitude, BRS, phase and coherence can be determined as a function of time. Using classical cross spectral analysis, one obtains only a single value for these parameters for each considered time period. In this paper, we consider the BP-IBI interaction as an open-loop system, i.e., the variation of IBI is due to BP fluctuations by means of the baroreflex control system.

We utilized the continuous wavelet transform from the MATLAB^®^ Wavelet Toolbox (MATLAB^®^ R2018b), which is both powerful and very user-friendly. We kept most MATLAB^®^ default settings, using Morse wavelets and four octaves with 12 steps each for the logarithmically distributed frequency values (49 frequencies). For our purpose, mainly the MATLAB^®^ cwt-function and the wcoherence-function were needed, for the one-dimensional wavelet transform and for the wavelet coherence and cross-spectrum, respectively. The wcoherence function was slightly modified to obtain non-normalized values for the wavelet cross spectrum.

The wBRS and the phase angle ϕ between systolic pressure and IBI were computed as follows:

wcsSS, wcsII, and wcsSI are the cross spectra of SAP vs. SAP, IBI vs. IBI, and SAP vs. IBI, respectively. For a registration with *N* beats, the dimension of these complex matrices is *N* × 49. Next, in matrix-notation:

$$\mathrm{wBRS}=\frac{\mathrm{abs}\,\left(\mathrm{wcsSI}\right)}{\mathrm{abs}\,\left(\mathrm{wcsSS}\right)}$$

ϕ=angle⁢(wcsSI)

$$r^{2}=\frac{\mathrm{abs}\,{\left(\mathrm{wcsSI}\right)}^{2}}{\mathrm{abs}\,\left(\mathrm{wcsSS}\right)\times \mathrm{abs}\,\left(\mathrm{wcsII}\right)}$$

This results in three matrices for wBRS, ϕ and *r*^2^, each with size *N* × 49. Values were discarded where *r*^2^ < 0.5, because for low coherence the wBRS and phase angle ϕ cannot be reliably estimated (De Boer et al., 1985; p. 353). For *N* beats, *N* × 2 values of intervals and pressure are given. Hence, the resulting data in the *N* × 49 matrices contain much dependency. For smoothing purposes, we used a moving average filter with width $\sqrt{N/2,}$ i.e., 32 for a recording of 2000 beats. The apparent frequency in the wavelet spectra is derived from the global sample rate, which is one over the averaged IBI. Because the *local* sample rate is one over the *local* IBI, this apparent frequency will vary with the *local* IBI (as will be visible in Figures 7A,B).

In order to study separately the low frequency 0.1 Hz-range and the higher frequency respiratory frequency range, wBRS-values and phase angle values were averaged in two octave-sized frequency-ranges: 0.07–0.15 Hz (LF) and 0.15–0.3 Hz (HF). Hence for the averaging over the frequencies, adjusted frequency ranges were used, with *f*_adj_= *f* × (IBI_local_/IBI_total_), where IBI_local_ is computed with a moving average filter with width $\sqrt{N/2}$.

### xBRS-Computation

We computed the instantaneous baroreflex sensitivity (xBRS) by the cross-correlation of blood pressure and interbeat interval (IBI) in a 10-s sliding window as described by Westerhof et al. (2004). In short: a 10 s window moves in 1-s steps over the SAP and IBI signals, and values are resampled at a 1 s rate after application of a cubic spline. Then, cross-correlations of SAP and IBI are computed in this window with 0, 1, 2, and 5 s delay. The delay with the highest cross-correlation value is taken as optimal delay τ. If this value is positive and significant at *p* < 0.05, the quotient of the standard deviations of IBI and of SAP is taken as the local xBRS value. For details see also Wesseling et al. (2017).

## Results

### Example for Subject A

**FIGURE 2:**
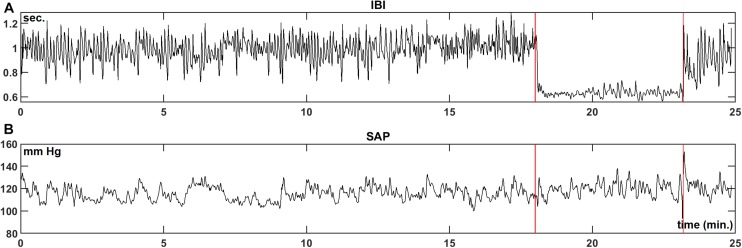
Example registration of IBI **(A)** and SAP **(B)** data for a resting supine subject, who is passively moved to a 70° head-up-tilt position and back (between the vertical lines). In this case, a large increase in heart-rate during HUT is observed, while the blood pressure level is hardly affected.

We present the analysis of the data for the first subject in our protocol. Figures 2A,B are the recordings of the IBI and SAP during the experimental procedure. The two vertical lines indicate the moments of the tilt up and the tilt down procedure, respectively. During the HUT-period, both the value and the variability of the IBI decrease, while little change is seen in the blood pressure values.

**FIGURE 3:**
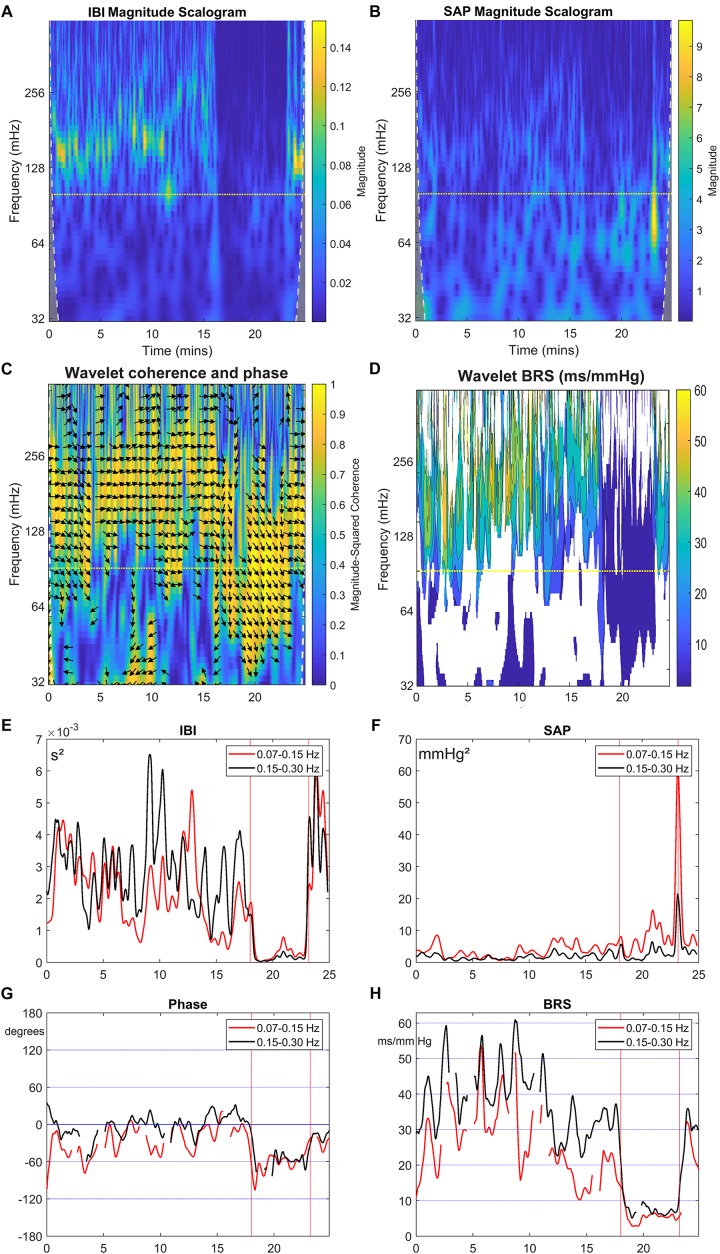
Wavelet analysis of IBI and SAP variability, and resulting BRS and phase between SAP and IBI for the data from Figure 2. The colors in the upper panels show the spectral power for each frequency and for each point of time, **(A)** for IBI and **(B)** for SAP. The 0.1 Hz frequency is indicated by a dotted line. **(C)** shows the wavelet coherence *r*^2^ (color) and phase (arrows) between SAP and IBI. Arrows pointing in the 3 o’clock indicate zero phase difference. Phase is shown only where *r*^2^ > 0.5. In **(D)**, the BRS is calculated from the data in the first two panels (only where *r*^2^ > 0.5). In **(E,F),** the mean spectral power in the LF band (red line) and in HF band (black line) is shown for IBI and SAP variability. **(G,H)** show the mean phase between SAP and IBI and the mean BRS for both frequency bands. A negative phase implies SAP-variations leading IBI-variations.

Figures 3A,B present the wavelet-power of the IBI- and SAP-data over the same period; vertical is the frequency in mHz, and the color in each point indicates the amount of spectral power at this frequency at this moment in time. The vertical color bar indicates the numeric values. A horizontal dotted line is drawn at frequency 0.1 Hz. In Figure 3A mainly respiratory influence is seen for the IBI-spectrum around the 0.18 Hz range, which disappears during the HUT period. The effect of a short episode of forced 0.1 Hz breathing at around 12 min is visible in the figure. The respiratory influence in the spectrum of SAP values (Figure 3B) is less clear, while some spectral contributions in the very low-frequency (<0.07 Hz) range may exist, but we focus in this paper on the higher frequencies. For this subject, a short burst of power is visible in the SAP spectrum around 23 min due to the tilt-down activity [Of note: this is not a movement artifact in the recording, but due to the cardiovascular dynamics during and after a fast tilt-down maneuver (Van Heusden et al., 2006)].

Next, Figure 3C presents the wavelet cross-spectrum of IBI and SAP, where the color indicates the value of the squared coherence *r*^2^. The arrows indicate the phase ϕ between IBI and SAP variations, but arrows are only drawn in time-frequency positions where *r*^2^ > 0.5. A horizontal arrow (“3 o’clock”) indicates no phase difference between IBI and SAP (ϕ = 0°), and an arrow pointing downward in the 6 o’clock direction implies SAP-variations to lead IBI-variations by 90° (ϕ = −90°), Figure 3C suggests during the supine period – until 18 min – mainly a ϕ = 0° value in the HF range, but during the HUT period (18–23 min), the phase is approximately ϕ = −60°. The same value of approximately ϕ = −60° is seen around 0.1 Hz under both experimental conditions, except in the case of paced breathing at that frequency (around 12 min, Figure 2). Note that a phase delay of −60° at a frequency of 0.1 Hz amounts to a delay of 1.7 s, or one and a half beat in the supine period, or almost three beats during HUT.

Figure 3D presents the computed wBRS for the various frequencies, where the values are indicated by the color bar. The wBRS values are shown only when *r*^2^ > 0.5. During the supine intervals the wBRS is seen to be in the 30–50 ms/mmHg range, while during HUT the wBRS is much lower and is more clearly defined at lower frequencies.

To differentiate between LF and HF variability, we present in Figures 3E–H plots of the various variables over time, averaged over the LF range (red line) and over the HF range (black line). In Figure 3E, the spectral powers of IBI for both frequency ranges are not too different, but large fluctuations are seen during the supine period. The power becomes much less during HUT. For the spectral values of SAP (Figure 3F), the power tends to increase during HUT. The spike at the end of the HUT period was discussed above.

During the supine period the phase between variations in IBI and SAP is around 0° for the HF band; its value fluctuates between 0° and around −60° for the LF band (Figure 3G). During HUT the phase is approximately −60° for both frequency bands. The wBRS-values (Figure 3H) show considerable variability during the supine period, with larger values of 30–45 ms/mmHg in the HF band than in the LF band (15–35 ms/mmHg). During HUT, the wBRS diminishes to values below 10 ms/mmHg.

### All Subjects

The same analysis was performed for the experimental recordings from all ten subjects. The relevant data are summarized in Table 1, where the subjects are ordered according to their supine IBI. Although variability exists between the results of the different subjects, supine, and HUT conditions are seen to produce highly different results. The paired two-tailed Student’s *t*-Test was applied to the results for the supine and HUT periods. As shown in Table 1, the differences between wBRS_supine_ and wBRS_HUT_ were found to be highly significant, both for the low and the high frequency range. Phase differences between the supine and HUT period were not significant for the LF, but highly significant for the HF.

**TABLE 1:** Summary of the results for the 10 subjects, ordered according to supine IBI.

													**wBRS**		**Phase**	
					**wBRS LF**		**wBRS HF**		**Phase LF**		**Phase HF**		**supine – HUT**		**supine – HUT**	
**Subject**	**IBI ± SD (s)**		**SAP (mmHg)**		**(ms/mmHg)**		**(ms/mmHg)**		**(degrees)**		**(degrees)**		**(ms/mmHg)**		**(degrees)**	
	**Supine**	**HUT**	**Supine**	**HUT**	**Supine**	**HUT**	**Supine**	**HUT**	**Supine**	**HUT**	**Supine**	**HUT**	**LF**	**HF**	**LF**	**HF**
J	0.64±0.04	0.59±0.04	129±4	147±7	8.1±2.7	4.7±1.2	12.3±3.0	5.7±1.4	−86±18	−77±26	−70±13	−76±12	3.4	6.6	–10	6
C	0.65±0.04	0.58±0.02	114±6	115±7	6.2±1.7	4.7±1.5	8.2±2.8	5.1±1.2	−59±18	−57±15	−58±19	−52±22	1.5	3.1	–3	–5
F	0.76±0.06	0.64±0.05	112±7	111±9	11.0±4.3	4.2±0.8	18.5±5.7	5.3±1.0	−41±20	−54±14	−32±30	−63±8	6.8	13.2	13	31
A	0.98±0.10	0.63±0.03	115±6	119±6	24.1±12.1	5.0±1.4	38.6±12.1	7.2±1.4	−35±38	−61±17	−4±23	−61±15	19.1	31.4	26	57
E	0.94±0.08	0.73±0.06	111±6	122±7	12.5±3.7	7.5±2.7	26.6±7.7	11.5±4.8	−83±21	−67±45	−46±15	−63±17	5.0	15.1	–15	17
G	0.99±0.08	0.75±0.06	102±6	106±7	14.6±4.9	7.3±1.6	19.8±5.6	7.9±1.7	−50±14	−62±12	−25±18	−70±10	7.3	11.9	12	45
H	1.04±0.03	0.82±0.03	102±4	118±8	8.0±3.2	4.2±1.1	11.9±3.7	4.3±1.1	−48±26	−65±16	−21±21	−71±16	3.8	7.6	17	50
I	1.02±0.07	0.87±0.06	117±7	117±6	13.0±5.6	8.8±3.7	24.9±7.7	14.7±4.1	−63±31	−74±18	−18±21	−18±15	4.2	10.2	11	–1
D	1.03±0.06	0.87±0.07	104±5	110±6	11.4±4.2	6.8±1.8	18.5±6.5	9.5±2.0	−46±38	−41±64	−33±20	−44±15	4.6	9.0	–5	11
B	1.31±0.10	0.95±0.08	113±5	113±6	24.4±12.8	9.5±2.3	47.4±13.6	14.1±4.2	−30±44	−34±12	4±20	−18±17	14.9	33.3	4	22
Mean ± SEM	0.94±0.06	0.74±0.04	112±3	118±4	13.3±2.0	6.3±0.6	22.7±3.9	8.5±1.2	−54±6	−59±4	−30±7	−54±7	7.1 ± 1.8	14.1 ± 3.2	5 ± 4	23 ± 7
												*p*-value:	0.003	0.002	0.250	0.008
													*	*	−	*

*For each subject, separate supine and HUT values (mean and standard deviation) are given for IBI, SAP, and wBRS and phase (SAP vs. IBI) for both frequency ranges. The last columns give the difference between mean wBRS and mean phase for supine and HUT conditions. The row indicated “Mean” shows the mean and standard error of the variables. The *p*-values for the paired two-tailed Student’s *t*-test are given.*

### wBRS-Variability

**FIGURE 4:**
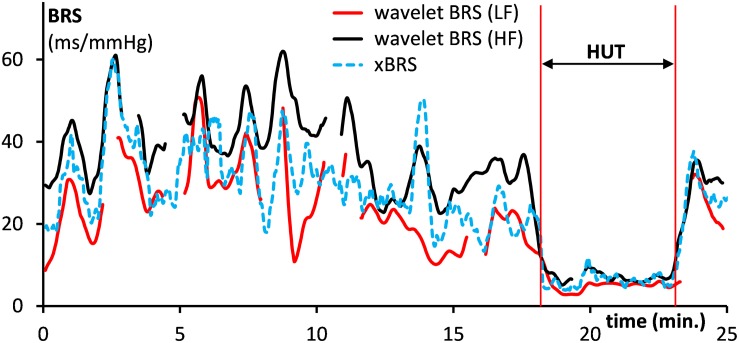
Comparison of the computed xBRS (dotted line) with the wavelet BRS in the LF range (drawn red line) and the HF range (black). Data are for the subject of Figure 2. The HUT period is indicated.

**FIGURE 5:**
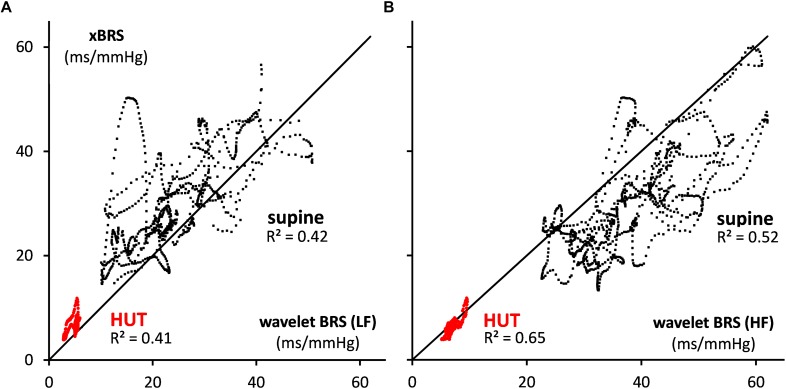
Scatterplots of xBRS vs. the wavelet BRS in the **(A)** LF and **(B)** HF range. The black dots indicate data points in supine position, and red dots are for HUT. The black line of identity is shown.

For comparison purposes, the data of Figure 2 were also analyzed by the sequential xBRS-method (Westerhof et al., 2004; Wesseling et al., 2017). Figure 4 shows the xBRS values together with the wBRS values for LF and HF from Figure 3H. The peaks and troughs in the signals of the xBRS-curve and the HF wBRS curve are rather similar, and the low BRS value during the HUT-maneuver is evident in all three curves. Figures 5A,B are scatterplots of xBRS vs. the LF and HF wBRS, respectively. Data are shown from the supine (black) and HUT (red) periods. A clear correlation between the values is seen between the xBRS and wBRS datasets, which have been computed using rather different methods.

### Simulated Data

**FIGURE 6:**
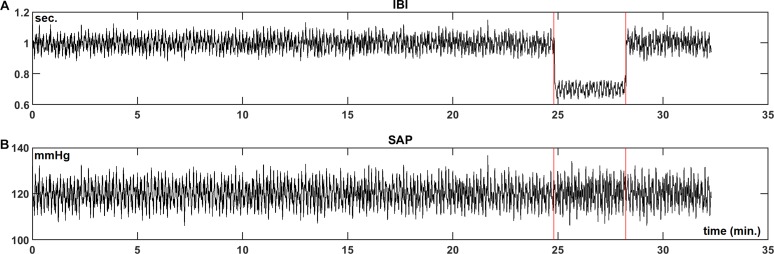
Simulated IBI **(A)** and SAP **(B)** data as described in section “Materials and Methods.” In the simulation, during the “HUT”-period (25 to 28.5 min) the mean IBI is decreased from 1.0 to 0.7 s and the vagal influence is reduced.

**FIGURE 7:**
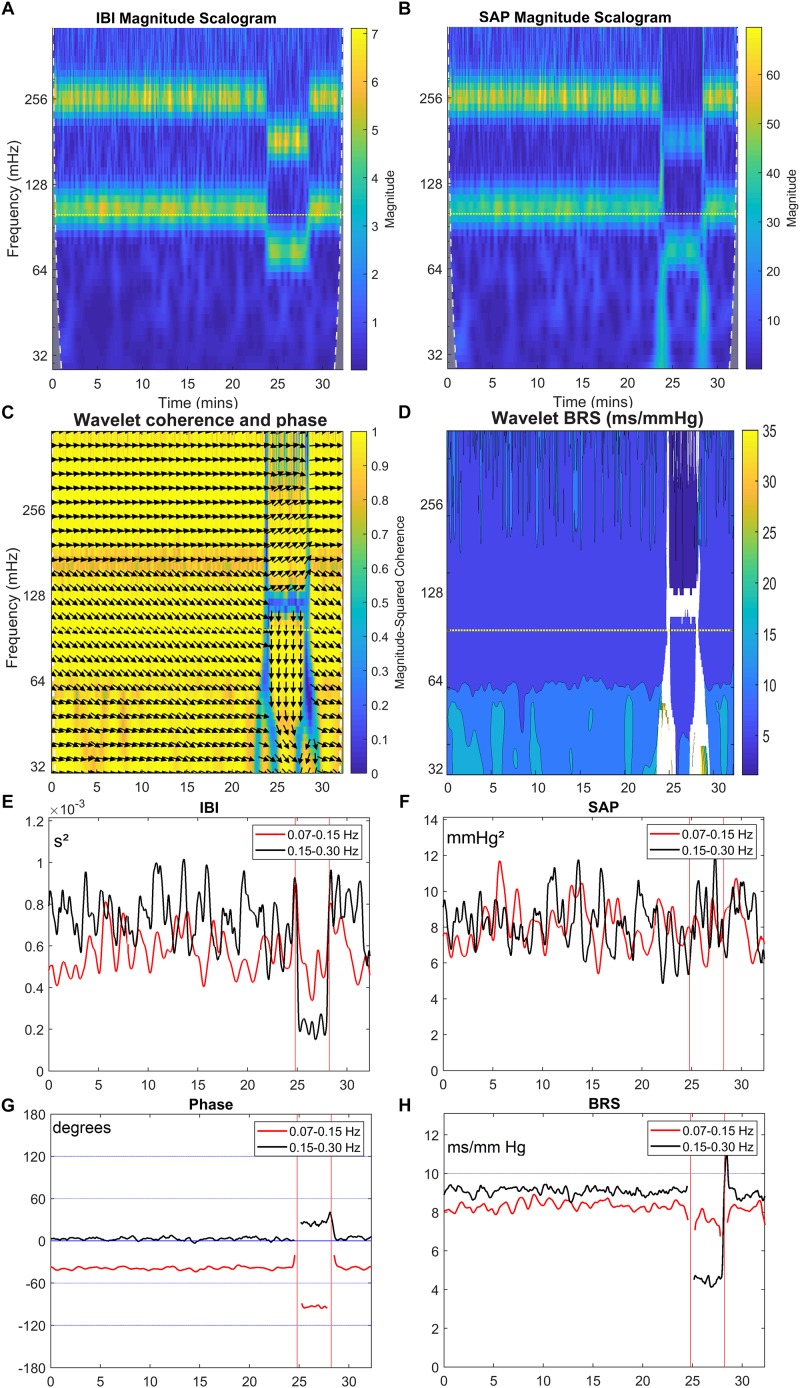
Wavelet analysis of IBI and SAP variability and resulting BRS for simulated data. The layout of this figure is identical to Figure 3. The colors in the upper panels show the spectral power for each frequency and for each point of time, **(A)** for IBI and **(B)** for SAP. **(C)** shows the wavelet coherence *r*^2^ (color) and phase (arrows) between SAP and IBI. In **(D)**, the BRS is calculated from the data in **(A,B)**. In **(E,F)**, the mean spectral power in the LF band (red line) and in HF band (black line) is shown for IBI and SAP variability. **(G,H)** show the mean phase between SAP and IBI and the mean BRS for both frequency bands. A negative phase implies SAP-variations leading IBI-variations. The imposed frequencies are 0.1 and 0.25 Hz but due to our beat-to-beat approach the *apparent* frequencies are slightly higher during the supine period, and lower during the (shorter) HUT period.

For the simulated IBI and SAP data (see section “Materials and Methods”), the same analysis was performed. Figures 6A,B show the IBI and SAP values, and Figures 7A,B present the wavelet spectral values as a function of time. The vertical axis shows the frequency as derived from a constant sample rate equal to one over the global mean of IBI (0.96 s in this simulation). However, with the wavelet technique results are obtained for frequencies related to the *local* sample rate, which is one over the *local* IBI (see Figure 6A): 1.0 s for beats 1–1500 and 1801–2000 (“supine”) and 0.7 s for beats 1501–1800 (“HUT”). Hence the *apparent* frequency in the wavelet spectra varies with the *local* IBI: the imposed 0.1 and 0.25 Hz frequencies appear at somewhat higher values during the supine period, and at lower values during HUT.

The wavelet cross spectrum in Figure 7C shows a high coherence, except at the moments of transition from supine to HUT and vice versa. Figures 7D–H show for the simulated data similar information as Figures 3D–H. Especially Figures 7G,H demonstrate that the wavelet analysis is well able to derive specific information from the SAP- and IBI-data. The various conditions (simulated supine vs. HUT) lead to quite different phase angles (Figure 7G) and wBRS-values (Figure 7H) in the two frequency-ranges. In the Appendix we show that these wavelet-derived values correspond well with the theoretical values as derived from the simulation parameters.

## Discussion

In this paper we apply a wavelet technique to analyze the cardiovascular regulation by the baroreflex during varying physiological conditions (supine and head-up-tilt). Using data from healthy subjects, we show that both the magnitude and the phase of the wBRS − being the wavelet derived BRS in open loop representation − differ between the supine condition and during a head-up-tilt maneuver. Analysis of simulated data demonstrates that the applied technique retrieves the correct parameters from the simulation (see Appendix). The main purpose of this paper is to describe the analysis technique and to demonstrate how the results can be applied to better understand the relations between cardiovascular signals. Therefore, we do not emphasize the interindividual differences between subjects nor the statistical characteristics of the method. This also applies to possible time patterns in the variability of the instantaneous wavelet results: in an earlier publication the 20–50 s variations in xBRS were attributed to the interaction of respiratory and cardiovascular control in the resting state. The present study was not designed to investigate this issue further.

Although several authors have used wavelet techniques in the study of cardiovascular regulation (e.g., Brychta et al., 2006; Stankovski et al., 2013; Singh et al., 2018), only few papers are known to us which applied cross wavelet techniques for the analysis of blood-pressure and heart-rate variability (Keissar et al., 2006, 2008, 2010; Kashihara et al., 2009). The first papers of Keissar et al. (2006, 2008) show principally the usefulness of this technique in the study of the ANS. In their 2010 paper these authors present the fluctuations of computed BRS values for supine subjects and during active standing up. In their registration the BRS values vary less rapidly compared to our results. We assume this to be due to the differences in analysis techniques. Kashihara et al. (2009) used wavelet techniques to identify the dynamic baroreflex properties from transient changes of step pressure inputs in anesthetized rabbits. Both Orini et al. (2010, 2012), Carrasco-Sosa and Guillen-Mandujano (2012) and Carrasco-Sosa and Guillén-Mandujano, 2013 used a different time-frequency analysis method [SPWVD: smoothed pseudo Wigner–Ville distribution (Xiang and Hu, 2012)] to dynamically assess the spontaneous BRS under varying physiological conditions. In our opinion, the wavelet approach has similar power as the SPWVD approach and is conceptually more straightforward. In addition, the wavelet computation is now readily available in the powerful MATLAB^®^ toolbox.

The summary of data presented in Table 1 indicates that BRS under supine conditions is higher than during the HUT period, both for the low and high frequency parts of the signals (Cooke et al., 1999). In the LF range, the mean phase difference between SAP to IBI was similar at −54° and −59° for the supine and HUT conditions, respectively. This corresponds well with the phase angle of around 70° as suggested by the 1987-model from De Boer et al. (1987). In accordance with this model, the phase for the HF variations was much lower (mean: −30°) for the data from the supine period; however, during HUT again a value of −54° was found, indicating a departure from the simple model.

We put the wBRS-technique to the test in two different ways. First, we compared the wBRS-results, separately for LF and HF, to the xBRS-method, which is a completely different technique to obtain a high rate of BRS-estimates in time (cf. Figures 4, 5). xBRS was shown to take a mid-position between LF-wBRS and HF-wBRS, which can be understood because xBRS is estimated in a sliding 10-s window and therefore both respiratory and 10-s variations will affect the resulting regression coefficient between BP and IBI. Figure 5B shows a good correlation between xBRS and HF-wBRS, with, as to be expected, consistently lower values for xBRS (Frederiks et al., 2000).

**FIGURE 8:**
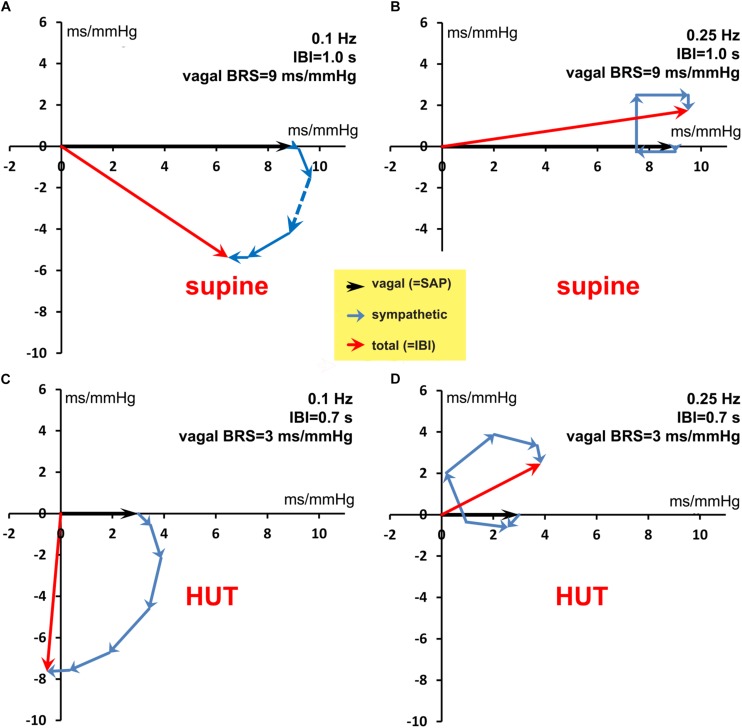
Vector addition of the frequency-dependent vagal and sympathetic BRS contributions for simulated data. The black vector indicates the vagal BRS contribution, coinciding in phase with the SAP variation. The blue vectors are the sympathetic BRS contributions, varying in size and direction, as suggested by Figure 1. The sum of vagal and sympathetic contributions determines the total BRS-vector (red), which controls the IBI-variations, the phase of which differs in general from the SAP phase. **(A,B)** are for the simulated supine conditions, with frequency 0.1 and 0.25 Hz, respectively. The orientation and size of the dashed vector in **(A)** is explained in detail in the text. **(C,D)** are for the HUT condition, with faster IBI and less vagal contribution. The figure shows that a variety of phase angles and BRS magnitudes can result from the combination of vagal and sympathetic effects.

As a second test, we constructed a set of simulated SAP data, which controlled the IBI data through simulated sympathetic and parasympathetic involvement. The Appendix shows how the computed wBRS values and the phase angles between variations in SAP and IBI can be well explained by the vector-addition of the vagal and sympathetic BRS-contributions (Figure 8 and Table 2). The Appendix also demonstrates that in the case of interaction of parasympathetic and sympathetic drive the apparent BRS-value can be quite different from expectation.

**TABLE 2:** Calculated length (BRS) and phase angle of the BRS-vectors, relating the IBI and SAP variations in our simulated data.

**Vector-summation**					
	**IBI**	**Vagal BRS**	**Freq.**	**BRS**	**Phase**
	**(s)**	**(ms/mmHg)**	**(Hz)**	**(ms/mmHg)**	**angle**
Supine	1.0	9	0.10	8.4	−40°
Supine	1.0	9	0.25	9.7	10°
HUT	0.7	3	0.10	7.6	−94°
HUT	0.7	3	0.25	4.6	33°
Supine	1.0	9	0.16	4.7	4°
HUT	0.7	3	0.16	3.2	172°

*The first two rows are with the simulation parameters for the “supine” period (Figures 8A,B) for the two imposed frequencies (0.1 and 0.25 Hz). The next two rows are for the “HUT” simulation (Figures 8C,D). In addition, values are given for an imposed frequency of 0.16 Hz (Figures 10A,B). In the latter case, under simulated HUT conditions the sympathetic influence dominates, leading to antiphase between IBI and SAP values.*

**FIGURE 9:**
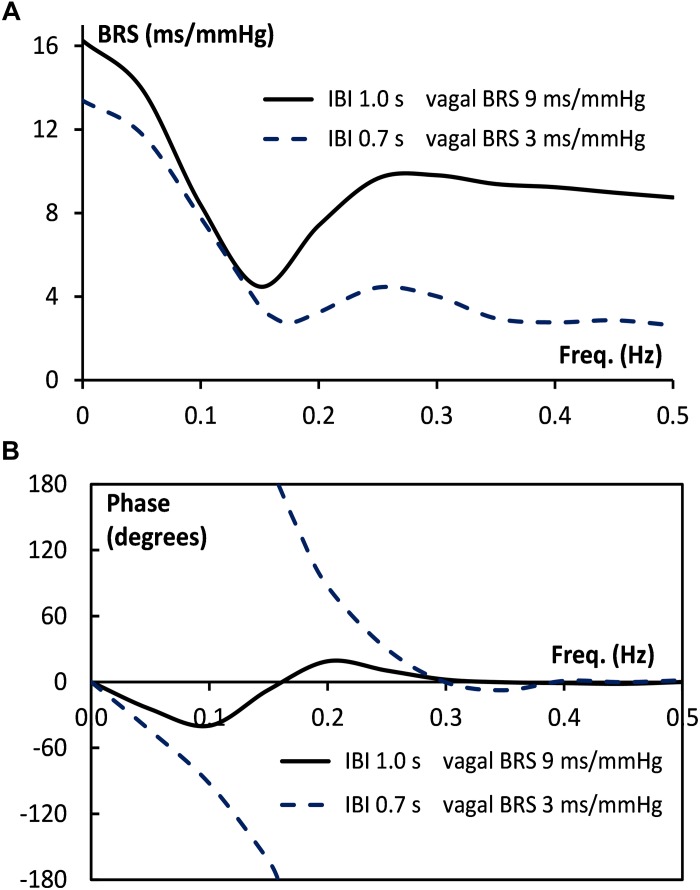
Bode plot of BRS for simulated supine and HUT data. The size **(A)** and the phase angle **(B)** of the BRS vector are given as a function of frequency, both for the supine (drawn line) and the HUT conditions (dotted line).

**FIGURE 10:**
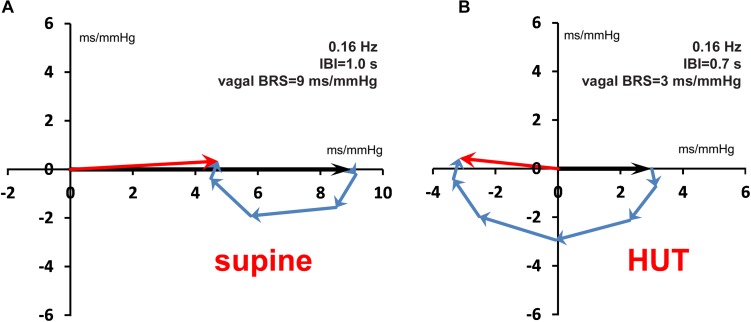
Vector addition of vagal and sympathetic BRS contributions for the 0.16 Hz frequency, both for the supine **(A)** and the HUT condition **(B)**. Around this frequency the Bode plot (Figure 9B) shows for the HUT condition a phase angle of ±180°. Note the antiphase between SAP and IBI variations in **(B)**.

We did not specifically analyze the short periods of paced breathing in the protocol; in particular the period of 6 breaths per minute is, generally, well-recognizable in IBI, not so much in BP (cf. Figure 2 at 12 min), therefore, it also appears clearly in the IBI spectrum (e.g., Figure 3A).

The large fluctuations in observed values, both for wBRS and xBRS, are notable. In earlier publications it has been argued that the blood pressure controlling system is working by noisy parameters, and hence large intrinsic variability results, even when time-averaged values are determined (Westerhof et al., 2004; Eckberg and Kuusela, 2005; Karemaker and Wesseling, 2008; Wesseling et al., 2017). Since our data come from resting, healthy subjects, the variability observed in this paper might be pointing directly at the inner working of what also is found by more abstract approaches such as entropy of the observed beat-to-beat values of IBI and BP (Richman and Moorman, 2000). The disappearance of variability after induction of anesthesia (Scheffer et al., 1993) is another argument why we consider the variability of wBRS and phase relations between BP and IBI to be real phenomena rather than the result of intricate calculations applied to inherently poor-quality, noisy data.

The system that regulates blood pressure and heart rate is a closed loop feedback system: blood pressure controls heart rate through the baroreflex, and the heart rate controls the blood pressure - to some extent - through diastolic runoff (Windkessel effect) and Starling’s law (De Boer et al., 1987; Faes et al., 2011; Porta et al., 2011). In ambulatory conditions, the separate determination of open- and closed loop gains is complex (Parati et al., 2019) and therefore often pharmacological methods are used for this purpose, applying Granger causality tests (Porta et al., 2013). A different approach to the study of causal couplings between the various cardiovascular parameters is in the information domain studies, often applying entropy measures (Faes et al., 2011; Javorka et al., 2017a, b, 2018). The technique we present in this paper only considers the baroreflex control of heart rate, which amounts to an open-loop analysis. This is justified if the feedforward gain of the baroreflex control is much larger than the feedback gain, each gain being scaled according to the noise entering the system at both ends of the loop (De Boer, 1985, p. 154). Although the observed phase-relationship between SAP- and IBI-variability that we present in this paper can be explained by a high baroreflex gain including sympathetic contributions, this result certainly does not yet prove that the baroreflex is the dominant factor in the cardiovascular loop.

In this paper we extended these earlier studies into the moment-to-moment variability of the condition of the autonomic nervous system (ANS). Heart rate- and blood pressure variability are considered to be reflections of the ANS, but most techniques to catch its condition in a number require extended periods of time. For instance, the recommendation of the “Task Force” (Malik et al., 1996) prescribes a period of at least 5 min to obtain stable measures. This shows the clash of requirements: on the one hand the cardiologist/clinician who needs a set number to describe a patient’s condition, a number that can show health or disease, and on the other hand the investigator/clinician who wants to look into the ANS and see how it changes and adapts to instantaneous stimuli, external or internal. In the latter case heart rate and blood pressure variability, and also the computed BRS, are only substitutes for what really is going on inside the central nervous system.

## Conclusion

The wavelet approach is an elegant way for time-frequency analysis of cardiovascular data. It enables the estimation of (cross)spectra and derived quantities such as wBRS during time-varying conditions without the need of arbitrary cut-offs. From the cross spectra, one obtains both the coherence and the phase between two signals, which can be used as a strict test for model-based studies. This gives a new way to manage, for instance, the data streams that are collected in intensive care clinical settings, but it may also point the way to a more appropriate use and interpretation of the multitude of e-health data which more and more people are collecting.

## Ethics Statement

The study used anonymized data from previous studies. These studies had been carried out in accordance with the recommendations of ESA’s Medical Review Board and the Medical Ethics Committee of the Academic Medical Center, Amsterdam. All subjects had given written informed consent in accordance with the Declaration of Helsinki. The protocol was approved by both Ethics Committees.

## Author Contributions

Both authors conceived and designed the study, analyzed and interpreted the data, and drafted the manuscript. JK acquired the data.

## Conflict of Interest Statement

The authors declare that the research was conducted in the absence of any commercial or financial relationships that could be construed as a potential conflict of interest.

## Appendix

### Explanation of the Cross Spectral Results for the Simulated Data

This appendix shows how the amplitudes and phase angles of the wBRS values as presented in Figures 7G,H can be derived from the time-course of the BRS as assumed in the simulations.

In our open-loop simulation, the varying SAP values affect the length of the successive intervals by the vagal contribution – acting on IBI without delay – and by the slower sympathetic contributions (Figure 1). The vagal BRS is taken as 9 and 3 ms/mmHg in the supine and HUT condition, respectively, and the strength of the sympathetic BRS is taken as increasing from zero to 3 ms/mmHg in the time span of 5.6–3.2 s before the considered IBI, and then decreasing to zero again at 0.8 s before the IBI. In the following calculation only small deviations of the mean values are considered and so the SAP and IBI values may be considered to be equally spaced at distances 1000 (“supine”) and 700 ms (“HUT”).

The wavelet analysis decomposes the fluctuations in SAP and IBI into their constituent frequencies, where the phase-angles between the SAP- and IBI-components may vary. Then, for every frequency the quotient of the IBI- and SAP-components results in a BRS-vector, which has a magnitude and phase angle [see also De Boer (1985, pp. 157–159)].

An example to manually compute the BRS from simulated data: consider Figure 8A, which represents 0.1 Hz fluctuations during the “supine” period, i.e., the mean IBI is taken as 1000 ms and the vagal contribution to the BRS is 9 ms/mmHg. Both the 0.1 Hz SAP-variation and the 0.1 Hz IBI-variation are presented as rotating vectors in this diagram. The phase of the 0.1 Hz SAP-variation is defined as 0° (direction of 3 o’clock in the figure) and rotation in this figure is anticlockwise. The variation in IBI is determined by a number of previous SAP values through the action of the baroreflex. In our simulation the vagal BRS contribution (no delay!) has also a phase of 0° and a strength of 9 ms/mmHg (heavy black arrow in Figure 8A). As to the sympathetic influence: the preceding SAP values have different weights and lags in their contribution to the total BRS (Figure 1). The sympathetic contribution of each SAP-value to the total BRS appears in Figure 8A as a vector (blue arrow).

For example: the dashed arrow indicates the baroreflex contribution of SAP*_n_*_–__3_ to IBI*_n_*. This SAP value leads the considered IBI by three beats or 3 s, which implies a phase angle of 3/10 × 360° = 108° for the considered 0.1 Hz contribution. The length of the dashed vector is given by the strength of the BRS-contribution at each instant, 2.75 ms/mmHg in this case. In a similar way the other sympathetic vectors are calculated. For a mean IBI value of 1.0 s, five previous SAP-values contribute to the sympathetic effect, with strengths 0.25, 1.5, 2.75, 2.0, and 0.75 ms/mmHg., and phase angles −36°, −72°, −108°, −144°, and −180°, respectively. These vectors are added to the vagal BRS (9 ms/mmHg) and the vector summation results in a total BRS (red arrow) of magnitude 8.4 ms/mmHg and a phase angle of −39.8°, which also represents the relative size and phase angle of the IBI-variation at this frequency.

The figure shows that interval variations lag behind pressure variations, as expected for a baroreflex-effect. The computed magnitude and phase angle values correspond with the wBRS results as shown in Figures 7G,H for the 0.1 Hz variations in the “supine” period.

In an identical manner, the resulting BRS-vectors for the HF band (Figure 8B) and for the “HUT” data (Figures 8C,D) are calculated (Table 2, top four lines). The results are always in agreement with the wavelet results from Figures 7G,H, although minor differences exist due to the presence of the added random noise in the simulated data.

The vector plots show that the delayed action of the sympathetic influence results in a frequency dependent BRS value, which needs not be in phase with the pressure variations. This is illustrated in Figure 9, which shows the Bode plot (magnitude and phase) for the frequency dependence of the BRS for our simulated data, both for the supine conditions (drawn line) and for the HUT period (dashed line). Figure 9A shows that under supine conditions (drawn line) a slightly different frequency of the “10-second rhythm,” for example 0.08 Hz or 0.12 Hz, results in quite different apparent BRS-values, 11 and 6 ms/mmHg, respectively. For the simulated HUT conditions, the phase plot (Figure 9B, dotted line) shows around 0.16 Hz a phase angle of ±180°, implying that for this frequency the interval variations are in antiphase with the pressure variations. Figure 10 shows the vector-plots of the BRS for this 0.16 Hz frequency: both for the supine and for the low-vagal-BRS HUT condition the sympathetic BRS-contribution is seen to counteract the vagal BRS action. Although this might be a questionable effect which exaggerates physiologically realistic conditions, the lag in the sympathetic effect can be expected to influence observed BRS values to a great extent.

## References

[B1] Bertinieri G. di Rienzo M. Cavallazzi A. Ferrari A. U. Pedotti A. Mancia G. (1985). A new approach to analysis of the arterial baroreflex. *J. Hypertens Suool.* 3 S79–S81. 2856787

[B2] Borst C. Karemaker J. M. (1983). Time delays in the human baroreceptor reflex. *J. Auton. Nerv. Syst.* 9 399–409. 10.1016/0165-1838(83)90004-8 6663021

[B3] Brychta R. J. Shiavi R. Robertson D. Biaggioni I. Diedrich A. (2006). A simplified two-component model of blood pressure fluctuation. *Am. J. Physiol. Heart Circ. Physiol.* 292 H1193–H1203. 10.1152/ajpheart.00645.2006 17012354 PMC1987355

[B4] Carrasco-Sosa S. Guillen-Mandujano A. (2012). “Correlation between spectral measures of systolic blood pressure variability and heart rate variability during paced breathing, standing and exercise,” in *Proceedings of the Computing in Cardiology*, (Krakow: IEEE), 169–172.

[B5] Carrasco-Sosa S. Guillén-Mandujano A. (2013). “Correlations between spectral measures of baroreflex sensitivity variability and hrv during supine position, paced breathing, standing and exercise,” in *Proceedings of the Computing in Cardiology*, (Zaragoza: IEEE),

[B6] Cooke W. H. Hoag J. B. Crossman A. A. Kuusela T. A. Tahvanainen K. U. O. Eckberg D. L. (1999). Human responses to upright tilt: a window on central autonomic integration. *J. Physiol.* 517 617–628. 10.1111/j.1469-7793.1999.0617t.x 10332107 PMC2269357

[B7] De Boer R. W. (1985). *Beat-to-beat blood-Pressure Fluctuations and Heart-Rate Variability in Man: Physiological Relationships, Analysis Techniques and a Simple Model.* Amsterdam: University of Amsterdam.

[B8] De Boer R. W. Karemaker J. M. Strackee J. (1984). Comparing spectra of a series of point events particularly for heart rate variability data. *IEEE Trans. Biomed. Eng.* 31 384–387. 10.1109/TBME.1984.325351 6745974

[B9] De Boer R. W. Karemaker J. M. Strackee J. (1985). Relationships between short-term blood-pressure fluctuations and heart-rate variability in resting subjects I: a spectral analysis approach. *Med. Biol. Eng. Comput.* 23 352–358. 10.1007/BF02441589 4046655

[B10] De Boer R. W. Karemaker J. M. Strackee J. (1987). Hemodynamic fluctuations and baroreflex sensitivity in humans: a beat-to-beat model. *Am. J. Physiol.* 253 H680–H689. 3631301 10.1152/ajpheart.1987.253.3.H680

[B11] De Boer R. W. Karemaker J. M. Van Montfrans G. A. (1986). “Determination of baroreflex sensitivity by spectral analysis of spontaneous blood pressure and heart rate fluctuations in man,” in *Neural Mechanisms and Cardiovascular Disease*, 3rd Edn, eds Lown B. Malliani A. Prosdocimi M. (Berlin: Springer-Verlag).

[B12] Eckberg D. L. Kuusela T. A. (2005). Human vagal baroreflex sensitivity fluctuates widely and rhythmically at very low frequencies. *J. Physiol.* 567 1011–1019. 10.1113/jphysiol.2005.091090 16002447 PMC1474223

[B13] Faes L. Nollo G. Porta A. (2011). Information domain approach to the investigation of cardio-vascular, cardio-pulmonary, and vasculo-pulmonary causal couplings. *Front. Physiol.* 2:80. 10.3389/fphys.2011.00080 22069390 PMC3209583

[B14] Frederiks J. Swenne C. A. TenVoorde B. J. Honzíkovaá N. Levert J. V. Maan A. C. (2000). The importance of high-frequency paced breathing in spectral baroreflex sensitivity assessment. *J. Hypertens.* 18 1635–1644. 10.1097/00004872-200018110-00015 11081777

[B15] Gisolf J. Akkerman E. M. Schreurs A. W. Strackee J. Stok W. J. Karemaker J. M. (2004). Tilt table design for rapid and sinusoidal posture change with minimal vestibular stimulation. *Aviat. Space Environ. Med.* 75 1086–1091. 15619866

[B16] Goldstein D. S. Horwitz D. Keiser H. R. (1982). Comparison of techniques for measuring baroreflex sensitivity in man. *Circulation* 66 432–439. 10.1161/01.CIR.66.2.432 7094250

[B17] Javorka M. Czippelova B. Turianikova Z. Lazarova Z. Tonhajzerova I. Faes L. (2017a). Causal analysis of short-term cardiovascular variability: state-dependent contribution of feedback and feedforward mechanisms. *Med. Biol. Eng. Comput.* 55 179–190. 10.1007/s11517-016-1492-y 27106759

[B18] Javorka M. Krohova J. Czippelova B. Turianikova Z. Lazarova Z. Javorka K. (2017b). Basic cardiovascular variability signals: mutual directed interactions explored in the information domain. *Physiol. Meas.* 38 877–894. 10.1088/1361-6579/aa5b77 28140353

[B19] Javorka M. Krohova J. Czippelova B. Turianikova Z. Lazarova Z. Wiszt R. (2018). Towards understanding the complexity of cardiovascular oscillations: insights from information theory. *Comput. Biol. Med.* 98 48–57. 10.1016/j.compbiomed.2018.05.007 29763765

[B20] Jevrejeva S. Moore J. C. Grinsted A. (2003). Influence of the arctic oscillation and el niño-southern oscillation (enso) on ice conditions in the baltic sea: the wavelet approach. *J. Geophys. Res.* 108:4677 10.1029/2003JD003417

[B21] Karemaker J. M. De Boer R. W. (2017). Vagal baroreflex latency in circulatory control. *J. Physiol.* 595 2197–2198. 10.1113/JP273766 28295368 PMC5350437

[B22] Karemaker J. M. Wesseling K. H. (2008). Variability in cardiovascular control: the baroreflex reconsidered. *Cardiovasc. Eng.* 8 23–29. 10.1007/s10558-007-9046-4 18041583

[B23] Kashihara K. Kawada T. Sugimachi M. Sunagawa K. (2009). Wavelet-based system identification of short-term dynamic characteristics of arterial baroreflex. *Ann. Biomed. Eng.* 37 112–128. 10.1007/s10439-008-9599-4 19003536

[B24] Kawada T. Sugimachi M. (2016). Open-loop static and dynamic characteristics of the arterial baroreflex system in rabbits and rats. *J. Physiol. Sci.* 66 15–41. 10.1007/s12576-015-0412-5 26541155 PMC4742515

[B25] Keissar K. Davrath L. R. Akselrod S. (2006). Time-frequency wavelet transform coherence of cardio-respiratory signals during exercise. *Comput. Cardiol.* 33 733–736.

[B26] Keissar K. Davrath L. R. Akselrod S. (2008). Wavelet transform coherence estimates in cardiovascular analysis: error analysis and feasibility study. *Comput. Cardiol.* 35 461–464. 10.1109/CIC.2008.4749078

[B27] Keissar K. Maestri R. Pinna G. D. La Rovere M. T. Gilad O. (2010). Non-invasive baroreflex sensitivity assessment using wavelet transfer function-based time-frequency analysis. *Physiol. Meas.* 31 1021–1036. 10.1088/0967-3334/31/7/011 20585147

[B28] Li K. Rüdiger H. Haase R. Ziemssen T. (2018). An innovative technique to assess spontaneous baroreflex sensitivity with short data segments: multiple trigonometric regressive spectral analysis. *Front. Physiol.* 9:10. 10.3389/fphys.2018.00010 29403393 PMC5786552

[B29] Malik M. Bigger J. T. Camm A. J. Kleiger R. E. Malliani A. Moss A. J. (1996). guidelines heart rate variability: standards of measurement, physiological interpretation, and clinical use. *Eur. Heart J.* 17 354–381. 10.1093/oxfordjournals.eurheartj.a014868 8737210

[B30] Orini M. Laguna P. Mainardi L. T. Bailón R. (2012). Assessment of the dynamic interactions between heart rate and arterial pressure by the cross time–frequency analysis. *Physiol. Meas.* 33 315–331. 10.1088/0967-3334/33/3/315 22354110

[B31] Orini M. Mainardi L. T. Gil E. Laguna P. Bailón R. (2010). “Dynamic assessment of spontaneous baroreflex sensitivity by means of time-frequency analysis using either RR or pulse interval variability,” in *Proceedings of the2010 Annual International Conference of the IEEE Engineering in Medicine and Biology Society*, Buenos Aires, 10.1109/IEMBS.2010.5626877 21096388

[B32] Parati G. Castiglioni P. Faini A. Di Rienzo M. Mancia G. Barbieri R. (2019). Closed-Loop cardiovascular interactions and the baroreflex cardiac arm: modulations over the 24 h and the effect of hypertension. *Front. Physiol.* 10:1–10. 10.3389/fphys.2019.00477 31133867 PMC6514241

[B33] Parati G. Di Rienzo M. Bertinieri G. Pomidossi G. Casadei R. Groppelli A. (1988). Evaluation of the baroreceptor-heart rate reflex by 24-hour intra-arterial blood pressure monitoring in humans. *Hypertension* 12 214–222. 10.1161/01.HYP.12.2.214 3410530

[B34] Parati G. Saul J. P. Di Rienzo M. Mancia G. (1995). Spectral analysis of blood pressure and heart rate variability in evaluating cardiovascular regulation: a critical appraisal. *Hypertension* 25 1276–1286. 10.1161/01.hyp.25.6.1276 7768574

[B35] Pickering T. G. Davies J. (1973). Estimation of the conduction time of the baroreceptor-cardiac reflex in man. *Cardiovasc. Res.* 7 213–219. 10.1093/cvr/7.2.213 4694105

[B36] Porta A. Bari V. Bassani T. Marchi A. Pistuddi V. Ranucci M. (2013). Model-based causal closed-loop approach to the estimate of baroreflex sensitivity during propofol anesthesia in patients undergoing coronary artery bypass graft. *J. Appl. Physiol.* 115 1032–1042. 10.1152/japplphysiol.00537.2013 23869064

[B37] Porta A. Catai A. M. Takahashi A. C. M. Magagnin V. Bassani T. Tobaldini E. (2011). Causal relationships between heart period and systolic arterial pressure during graded head-up tilt. *Am. J. Physiol. Regul. Integr. Comp. Physiol.* 300 R378–R386. 10.1152/ajpregu.00553.2010 20962207

[B38] Richman J. S. Moorman J. R. (2000). Physiological time-series analysis using approximate entropy and sample entropy. *Am. J. Physiol. Heart Circ. Physiol.* 278 H2039–H2049. 10843903 10.1152/ajpheart.2000.278.6.H2039

[B39] Robbe H. W. J. Mulder L. J. M. Ruddel H. Langewitz W. A. Veldman J. B. P. Mulder G. (1987). Assessment of baroreceptor reflex sensitivity by means of spectral analysis. *Hypertension* 10 538–543. 10.1161/01.hyp.10.5.538 3666866

[B40] Rua A. Nunes L. C. (2009). International comovement of stock market returns: a wavelet analysis. *J. Empir. Finance* 16 632–639. 10.1016/J.JEMPFIN.2009.02.002

[B41] Rudas L. Pflugfelder P. W. Kostuk W. J. (1993). Immediate cardiovascular responses to orthostasis in the early and late months after cardiac transplantation. *Int. J. Cardiol.* 38 141–150. 10.1016/0167-5273(93)90173-E 8454376

[B42] Scheffer G. J. TenVoorde B. J. Karemaker J. M. Ros H. H. De Lange J. J. (1993). Effects of thiopentone, etomidate and propofol on beat-to-beat cardiovascular signals in man. *Anaesthesia* 48 849–855. 10.1111/j.1365-2044.1993.tb07412.x 8238825

[B43] Singh R. S. Saini B. S. Sunkaria R. K. (2018). Times varying spectral coherence investigation of cardiovascular signals based on energy concentration in healthy young and elderly subjects by the adaptive continuous morlet wavelet transform. *IRBM* 39 54–68. 10.1016/j.irbm.2017.12.004

[B44] Smyth H. S. Sleight P. Pickering G. W. (1969). Reflex regulation of arterial pressure during sleep in man: a quantitative method of assessing baroreflex sensitivity. *Circ. Res.* 24 109–121. 10.1161/01.RES.24.1.109 4303309

[B45] Stankovski T. Cooke W. H. Rudas L. Stefanovska A. Eckberg D. L. (2013). Time-frequency methods and voluntary ramped-frequency breathing: a powerful combination for exploration of human neurophysiological mechanisms. *J. Appl. Physiol.* 115 1806–1821. 10.1152/japplphysiol.00802.2013 24114700 PMC3882935

[B46] Torrence C. Compo G. P. (1998). A practical guide to wavelet analysis. *Bull. Am. Meteorol. Soc.* 79 61–78.

[B47] Van Heusden K. Gisolf J. Stok W. J. Dijkstra S. Karemaker J. M. (2006). Mathematical modeling of gravitational effects on the circulation: importance of the time course of venous pooling and blood volume changes in the lungs. *Am. J. Physiol. Heart Circ. Physiol.* 291 H2152–H2165. 10.1152/ajpheart.01268.2004 16632542

[B48] Wesseling K. H. Karemaker J. M. Castiglioni P. Toader E. Cividjian A. Settels J. J. (2017). Validity and variability of xBRS: instantaneous cardiac baroreflex sensitivity. *Physiol. Rep.* 5:e135091. 10.14814/phy2.13509 29180481 PMC5704083

[B49] Westerhof B. E. Gisolf J. Stok W. J. Wesseling K. H. Karemaker J. M. (2004). Time-domain cross-correlation baroreflex sensitivity: performance on the EUROBAVAR data set. *J. Hypertens.* 22 1371–1380. 10.1097/01.hjh.0000125439.28861.ed 15201554

[B50] Xiang L. Hu A. (2012). Comparison of methods for different timefrequency analysis of vibration signal. *J. Softw.* 7 68–74. 10.4304/jsw.7.1.68-74

